# Recent Progress in Electrolytes for Zn–Air Batteries

**DOI:** 10.3389/fchem.2020.00372

**Published:** 2020-05-26

**Authors:** Peng Chen, Keyi Zhang, Dejian Tang, Weilin Liu, Fancheng Meng, Qiuwei Huang, Jiehua Liu

**Affiliations:** Future Energy Laboratory, School of Materials Science and Engineering, Hefei University of Technology, Hefei, China

**Keywords:** Zn–air battery, electrolyte, alkaline electrolytes, room temperature ionic liquid, quasi-solid flexible electrolyte

## Abstract

Zn–air battery is considered as one of the most promising candidates for next-generation batteries for energy storage due to safety, high energy density, and low cost. There are many challenges in electrolytes for developing high-performance rechargeable Zn–air cells as well as electrocatalysts. An electrolyte is the crucial part of the rechargeable Zn–air batteries that determine their capacity, cycling stability, and lifetime. This paper reviews the most recent progress in designing and fabricating electrolytes in aqueous and flexible Zn–air batteries. The discussion on the surface reaction relationships was covered between air–catalyst–electrolyte and electrolyte–zinc reaction mechanism. We highlight the recent developments of three different electrolytes in zinc–air battery: aqueous electrolyte, room temperature ionic liquid, and quasi-solid flexible electrolyte. Furthermore, the general perspective is proposed for designing and fabricating electrolytes to improve the performance and prolong the lifetime of Zn–air batteries.

## Introduction

Zn–air battery has high specific energy (1,218 Wh·kg^−1^). Meanwhile, its inherent features, including safety and lower cost, make it one of the most promising next-generation batteries (Fu et al., 2017; Tan et al., 2017; Han et al., 2019). The role of electrolytes has been overlooked compared to the hot research on bifunctional air electrodes for Zn–air batteries. The performance of electrolytes directly determines the ionic conductivity and interfacial properties of the Zn–air battery in operation. Furthermore, it further affects the capacity, cycling stability, and charging and discharging efficiency of the cell (Pei et al., 2014). Zn–air batteries are developing toward high efficiency and durability, which cannot separate from the support of electrolyte with excellent performance in all aspects (R. Mainar et al., 2016). Thus, it is urgently significant to delve into the workings of electrolytes in Zn–air batteries (Mainar et al., 2018).

At present, the alkaline electrolyte is still widely used in zinc-based batteries to meet the requirements of low cost and high ionic conductivity and ensure the stability of the zinc electrode (R. Mainar et al., 2016; Xu et al., 2020). However, it is susceptible to environmental CO_2_ and relative humidity. Zn–air battery is mainly dependent on the performance of the air electrode. Unfortunately, CO_2_ can lead to the generation of K_2_CO_3_ in the electrolyte, which adversely affects the void in the air electrode (Wang et al., 2014; Fu et al., 2017). Zn–air batteries must solve the problem of evaporation of electrolyte or absorption of water from the external environment to work well in the complex external environment. The former makes the battery expand, and the latter affects the transfer of OH^−^ (Chakkaravarthy et al., 1981; Mainar et al., 2018). Room temperature ionic liquids (RTILs), and solid electrolytes are alternative and effective solutions to solve the above problem. However, their performance was limited by their low ionic conductivity and unqualified interface. Therefore, in the following parts, we will discuss the research status of alkaline electrolytes and non-aqueous electrolytes in Zn–air batteries.

## Aqueous Electrolyte

LiOH, NaOH, and KOH are the common electrolytes for Zn–air batteries. Compared with neutral and acidic electrolytes, the alkaline electrolytes are well-matched with zinc electrodes and catalyst materials. Meanwhile, there is high ionic conductivity and low viscosity in KOH electrolyte. When Zn–air battery discharges, the external oxygen enters the battery and reacts (Equation 1) (oxygen reduction reaction) at the gas–liquid–solid interface (oxygen, electrolyte, electrocatalyst). The zinc electrode transports electrons to the air electrode through the external load, and the OH^−^ at the reaction site generates ${\text{Zn(OH)}}_{4}^{2-}$ (Equation 2). When the concentration of ${\text{Zn(OH)}}_{4}^{2-}$ reaches the maximum, it will further decompose into ZnO (Equation 3). The complete reaction of the zinc electrode is shown in Equation 4. During the charging process, backward reaction (Equation 1) (oxygen evolution reaction) is performed at the zinc–electrolyte interface, and electrical energy is stored, while zinc deposits by backward reaction (Equation 3).

(1) $$\begin{array}{l} {\text{O}}_{2}+2{\text{H}}_{2}\text{O}+4{\text{e}}^{-}↔4\text{O}{\text{H}}^{-}\text{E}=0.40\text{V vs}.\text{SHE} \end{array}$$

(2) $$\begin{array}{l} \text{Zn}+4\text{O}{\text{H}}^{-}↔{\text{Zn}\left(\text{OH}\right)}_{4}^{2-}+2{\text{e}}^{-}\text{E}=1.26\text{V vs}.\text{SHE} \end{array}$$

(3) $$\begin{array}{l} {\text{Zn}\left(\text{OH}\right)}_{4}^{2-}↔\text{ZnO}+{\text{H}}_{2}\text{O}+2\text{O}{\text{H}}^{-} \end{array}$$

(4) $$\begin{array}{l} \text{Zn}+2\text{O}{\text{H}}^{-}↔\text{ZnO}+{\text{H}}_{2}\text{O}+2{\text{e}}^{-}\text{E}=1.26\text{V vs}.\text{SHE} \end{array}$$

When the concentration of KOH is 6 M, the current exchange density of Zn/Zn^2+^ reaches 0.21 A cm^−2^, and the solubility of ZnO increases with the concentration of KOH (See and White, 1997; Dyer et al., 2009). Therefore, we must pay attention to the adverse effect of high concentration KOH electrolyte on the zinc electrode. The high concentration of ZnO produces excess ${\text{Zn(OH)}}_{4}^{2-}$ and precipitates after the discharge, which increases the passivation resistance of the zinc electrode. Besides, the oxygen reduction kinetic parameters of zinc were very high, resulting in the dissolution, migration, and redeposition of zinc under various conditions (R. Mainar et al., 2016).

There are two main strategies to solve this problem. One is to change the composition and structure of the zinc electrode, and the other is to find the appropriate electrolyte additives. Reported methods such as making the zinc electrode have a 3-D structure (Parker et al., 2014; Chamoun et al., 2015; Yan et al., 2015) or the efficient additive for the zinc electrode (Fan et al., 2013; Masri and Mohamad, 2013; Huang et al., 2015) have proven to be an effective solution strategy. It is an urgent task to accurately measure the potential and concentration of zinc ions on the surface of the zinc electrode to provide adequate theoretical support for improving the living conditions of the zinc electrode in the alkaline electrolyte. In Table 1, we summarized the recent work on alkaline electrolyte additives. Suitable additives in electrolytes can improve the shape change of the zinc electrode and the performance of the Zn–air battery. If we can reduce the concentration of KOH as far as possible without affecting the ionic conductivity of the electrolyte, we believe that the performance of the Zn–air battery will be further improved. By adding K_2_CO_3_ to high-concentration KOH solution and optimize the structure of the battery, Schröder et al. (2015) not only obtained stable electric potential but also improved the actual energy density and long-term stability of the Zn–air battery. Besides, the inhibition of dendrite growth and hydrogen evolution of zinc electrode is also reported in Zn–air battery with the alkaline electrolytes using sodium dodecylbenzene sulfonate (SDBS) (Yang et al., 2004), polyethylene glycol (PEG) (Banik and Akolkar, 2013), tartaric/succinic/citric acid (Lee et al., 2006), and tetra-alkyl ammonium hydroxides (Lan et al., 2007).

**Table 1:** Summary of recently reported alkaline electrolyte additive for Zn–air batteries.

**Electrolyte composition**	**Electrode materials**	**Specific capacity**	**Power density**	**Durability**	**References**
6 M KOH + 0.2 M zinc acetate	Zinc plate//Co–Co_3_O_4_@NAC@NF	721 mAh ${\text{g}}_{\text{Zn}}^{-1}$ @10 mA cm^−2^	164 mW cm^−2^ @0.63 V	35 h@10 mA cm^−2^ for 20 min per cycle	Zhong et al., 2020
6 M KOH + 0.2 M zinc acetate	Zinc foil//Co_3_O_4−x_@CP	800 mAh ${\text{g}}_{\text{Zn}}^{-1}$ @5 mA cm^−2^	122 mW cm^−2^ @230 mA cm^−2^	150 h@5 mA cm^−2^ for 20 min per cycle	Li et al., 2020
6 M KOH + 0.2 M ZnCl_2_	Zinc plate//Pt–SCFP@CC	781 mAh ${\text{g}}_{\text{Zn}}^{-1}$ @10 mA cm^−2^	122 mW cm^−2^ @214 mA cm^−2^	80 h@5 mA cm^−2^ for 20 min per cycle	Wang et al., 2020
7 M KOH + 5–20% v/v DMSO	Zinc granules//MnO_2_@NF	550 ${\text{mAhg}}_{\text{Zn}}^{-1}$ @10 mA cm^−2^	130 mW cm^−2^ @150 mA cm^−2^	600 cycles@discharge@75 mA cm^−2^ and charge@25 mA cm^−2^	Hosseini et al., 2019
8 M KOH + 0–50% v/v ethanol	Zinc granules//MnO_2_@NF	470 mAh ${\text{g}}_{\text{Zn}}^{-1}$ @25 mA cm^−2^	32 mW cm^−2^ @30 mA cm^−2^	N/A	Hosseini et al., 2018
100 ml 1 M KOH + 0.1 g water-suspended graphene	Zinc strip//Co–Sn–CNP@SS	212.6 mAh ${\text{g}}_{\text{Zn}}^{-1}$ @1 mA cm^−2^	N/A	Discharge 15 h@1 mA cm^−2^	Kumar et al., 2019

*NF, Ni foam; CP, carbon paper; CC, carbon cloth; SS, stainless steel*.

Zn–air battery is a semi-open system that requires the rich oxygen from the outer environment to participate in the reaction process. Carbon dioxide (CO_2_) is difficult to avoid in a moist atmosphere. CO_2_ from the outer atmosphere enters the battery through the air electrode and reacts with OH^−^ in the electrolyte (Equations 5, 6).

(5) $$\begin{array}{l} \text{C}{\text{O}}_{2}+\text{O}{\text{H}}^{-}\to \text{HC}{\text{O}}_{3}^{-} \end{array}$$

(6) $$\text{HC}{\text{O}}_{3}^{-}+\text{O}{\text{H}}^{-}↔\text{C}{\text{O}}_{3}^{2-}+{\text{H}}_{2}\text{O}$$

The ionic conductivity of the electrolyte becomes weakened due to the generation of ${\text{HCO}}_{3}^{-}$ and ${\text{CO}}_{3}^{2-}$ and the low solubility of K_2_CO_3_ and KHCO_3_. When they deposit on the air electrode, the oxygen transfer will be blocked to some extent, resulting in the performance decline of the Zn–air battery. Optimizing the structure of the Zn–air battery and the composition of the gas adsorption layer to allow oxygen to pass through unimpeded but to inhibit the passage of carbon dioxide and water vapor is an ideal solution. To solve the above problems, investigators also put forward several solutions. Pedicini et al. (1996) set up an air manager system for recirculating reactant air in the metal–air battery. Goldstein et al. (1997) put forward a scrubber system for removing carbon dioxide from a metal–air or fuel cell battery. Pedicni (2002) proposed to limit carbon dioxide and water vapor when the battery is not in use by loading a responsive air door for an electrochemical cell. There are many solutions to solve these problems, but the limitations are high-cost thresholds and reduced space utilization, which limit the development of Zn–air batteries in practical applications.

The flow electrolyte system is a very effective method for Zn–air batteries. The electrolyte is pumped and circulated through a power system of external pipes and pumps. In addition to removing the precipitated carbonate and other by-products through external filters, the flowing electrolyte improves OH^−^ transfer and reduces concentration gradients (Iacovangelo and Will, 1985; Cheng et al., 2007). Compared with the static electrolyte, Zn–air battery is much improved including the cycle life and operating voltage with a circulating electrolyte system. However, the power of electrolyte circulation needs to be supported by an external pumping system and electric energy. Therefore, if the electrolyte circulation system is put into practical application, it is necessary to solve the problem that it is difficult to apply to the large-scale grid energy storage system with strict space and weight requirements.

## Room Temperature Ionic Liquid

The room temperature ionic liquid is a molten salt that exists as a liquid at or below room temperature. It has a wide electrochemical window and is not easily ignited (Balaish et al., 2014). Therefore, more and more attention was paid to RTILs as substitutes for alkaline electrolytes. The inherent safety and stability of RTILs over a wide range of electrochemical potentials have led to its application in lithium-based batteries (Chou et al., 2008; Xiang et al., 2010). The use of RTILs in Zn-air batteries can effectively solve the problems of zinc electrode damage (Simons et al., 2012), CO_2_ damage, and electrolyte evaporation (Harting et al., 2012) in the alkaline electrolyte of the water system mentioned above, and make it possible for the battery to work at high temperatures. Moreover, for aprotic RTILs, the absence of protons can effectively avoid the corrosion of the zinc electrode caused by hydrogen evolution. Therefore, RTILs, as an electrolyte for Zn–air batteries, have put on the list in recent years.

RTILs used as an electrolyte for Zn–air cell, zinc oxidize to Zn^2+^ during discharge, and the reversible electrochemical reaction of zinc in RTILs has proven to be feasible (Xu et al., 2015). What we need to note here is that inappropriate RTILs may form insoluble substances with Zn^2+^ and make them unable to be reduced effectively. The mechanism of air electrode in RTIL electrolyte was proposed (Kar et al., 2014).

When oxygen reduction occurs in the RTILs electrolyte, oxygen gains electrons and forms a superoxide (${\text{O}}_{2}^{\cdot -}$) (Equation 7). This reaction is considered quasi-reversible (AlNashef et al., 2002). For aprotic RTILs, there is no further electron transfer due to the presence of superoxide. In contrast, for protic RTILs, superoxide is a strong nucleophile that can further react with protons in RTILs to form per-hydroxy radical (${\text{HO}}_{2}^{\cdot }$) (Equation 8). Then, per-hydroxy radical can also react with superoxide to form peroxide (${\text{HO}}_{2}^{-}$) (Equations 9, 10) and finally complete the reduction process (Equation 11).

(7) $${\text{O}}_{2}+{\text{e}}^{-}\to {\text{O}}_{2}^{\cdot -}$$

(8) $${\text{O}}_{2}^{\cdot -}+{\text{H}}^{+}\to \text{H}{\text{O}}_{2}^{\cdot }$$

(9) $$\text{H}{\text{O}}_{2}^{\cdot }+{\text{O}}_{2}^{\cdot -}\to \text{H}{\text{O}}_{2}^{-}+{\text{O}}_{2}$$

(10) $$\text{H}{\text{O}}_{2}^{\cdot }+{\text{e}}^{-}\to \text{H}{\text{O}}_{2}^{-}$$

(11) $$\text{H}{\text{O}}_{2}^{-}+{\text{H}}^{+}\to {\text{H}}_{2}{\text{O}}_{2}$$

As for whether hydrogen peroxide can further decompose into H_2_O, Zeller (2011) points out that it is determined by the electrode used. According to Kar et al. (2014)'s summary of oxygen reduction and oxygen precipitation reactions in RTILs, in the reaction, as mentioned above, paths have proven to be reversible and relatively stable peroxide products. However, there are still some related disproportionation reactions. Hydrogen peroxide requires less activation energy to produce oxygen, which makes it an effective support for oxygen reduction, and oxygen evolution reactions in RTILs.

The development of RTILs in the Zn–air battery still faces enormous challenges. On the one hand, the high cost of RTILs makes it challenging to use on a large scale. On the other hand, the RTILs' dual-electron reaction mechanism reduces the energy density of the battery, coupled with its high viscosity and low conductivity, which means that the Zn–air battery can only operate at a low current. With Li_0.87_Na_0.63_K_0.50_CO_3_ and NaOH as the electrolyte, Liu et al. (2017) investigated the Zn–air battery able to charge and discharge at 550°C for 100 cycles with Coulombic efficiency of 96.9%. When Ingale et al. (2017) applied diethylmethylammonium trifluoro-methanesulfonate (DEATfO) ionic liquid to the Zn–air battery, they found that although there was no zinc dendrite generation, the weak surface tension of DEATfO resulted in unsatisfactory energy density (Pozo-Gonzalo et al., 2014). Furthermore, Ghazvini et al. (2018) pointed out the positive effect of water addition on ionic interaction when RTIL electrolyte was used in Zn–air batteries. The above work provides a good strategy for improving the performance of the Zn–air battery with RTILs as an electrolyte.

Further, the application of more types of RTILs in Zn–air batteries should be investigated, including the beneficial effects of additives in RTILs. It is also necessary to develop specific bifunctional catalysts to reduce the energy barrier of oxygen reduction reaction and oxygen evolution reaction. Although the RTIL electrolyte needs further study in terms of interface properties, the electrochemical reaction mechanism of oxygen and the migration path of active substances, various pieces of evidence indicate that RTILs are the promising electrolytes for Zn–air batteries.

## Quasi-Solid Flexible Electrolyte

With the increasing demand for flexible wearable electronic devices, the research on flexible batteries, especially quasi-solid electrolytes, has put forward higher requirements. Compared with other metal–air batteries, Zn–air batteries with high volume energy density have the characteristics of low cost and high safety. In contrast, zinc as an electrode has more energetic mechanical properties and productivity in flexible batteries. For example, Zn–MnO_2_ batteries using polymer electrolytes were commercially produced using printing technology (MacKenzie and Ho, 2015). Therefore, it is necessary to carry out scientific research on the structure and performance of a flexible Zn–air battery, and the production of this type of battery and the matching quasi-solid electrolyte needs to be continuously optimized.

The quasi-solid flexible electrolyte is usually prepared from alkaline aqueous solution and polymers such as polyvinyl alcohol (PVA) (Fan et al., 2019), polyacrylic acid (PAA) (Wu et al., 2006; Zhu et al., 2018), gelatin (Park et al., 2015), and related graft copolymer (Yu et al., 2017), which are required to meet the stable configuration, cathode and anode separation, and qualified ionic conductivity. During the preparation process, most of the quasi-solid flexible electrolytes can form a cross-linked network with a large number of hydrophilic functional groups (such as hydroxyl groups), which enables higher water retention and ionic conductivity in quasi-solid flexible electrolytes. In primary Zn–air cell, alkaline gel electrolyte can effectively reduce the leakage and volatilization of electrolyte and has been applied (Hilder et al., 2009). However, for rechargeable flexible Zn–air batteries, on account of the zinc electrode in the quasi-solid flexible electrolyte, they can only carry a small amount of ${\text{Zn(OH)}}_{4}^{2-}$. The process of ZnO reduction to ${\text{Zn(OH)}}_{4}^{2-}$ is blocked (Xu et al., 2015). Therefore, it is a big challenge to realize rechargeable Zn–air batteries to work in a large current.

Flexible power density and cycle performance of the Zn–air battery have been highly favored. However, there are several important aspects in the bifunctional catalyst for electrochemical oxygen reactions, the ionic conductivity of the quasi-solid flexible electrolyte, and the performance of the electrolyte–electrode interface. The ionic conductivity of the electrolyte depends mainly on the type of polymer and electrolyte additives. Fan et al. (2019) prepared a porous PVA + SiO_2_ electrolyte with high ionic conductivity of 57.3 mS cm^−1^ and excellent cycling performance and power density. Li et al. (2019) fabricated the polymer dielectric TEAOH-PVA, which still had the ionic conductivity of 30 mS cm^−1^ after 2 weeks, showing excellent service and working life. It is not difficult to find that a single polymer can hardly become a flexible electrolyte of a quasi-solid state with excellent performance. However, a small number of additives can significantly improve the performance of electrolytes, which is also a process of polymer functionalization. This is mainly because the additive optimizes the structure of the crosslinked network of the polymer electrolyte, increases the number of hydrophilic functional groups (such as hydroxyl groups), and further improves water retention ability of the electrolyte, which has a great influence on the ionic conductivity. Moreover, in addition to the ionic conductivity and water retention performance of the quasi-solid flexible electrolyte, the transfer rate of OH^−^ and ${\text{Zn(OH)}}_{4}^{2-}$ should also be put to more attention, which has been paid insufficient emphasis at present. Their transfer process also has a profound impact on the energy density and other performance of flexible Zn–air batteries.

There is a challenge to improve the performance of the electrolyte–electrode interface (especially the electrolyte–air electrode interface) in the flexible Zn–air battery. The wettability of the quasi-solid flexible electrolyte was reduced, which makes it much more difficult for the catalyst to perform its function than in the alkaline electrolyte of the water system. When assembling the battery, Xu et al. (2019) pressed the battery for 3 min under 3 MPa through a tablet press to make the laminated structure more complete, and the flexible Zn–air battery could stabilize the circulation for 35 h. More exploration is still needed to improve the electrolyte–electrode interface, the preparation of electrolyte, and the battery packaging method.

The flexible Zn–air battery also puts forward to the higher requirements on the bending, stretching, and compression performance of the zinc electrode, air electrode, and electrolyte in the battery. The flexible Zn–air battery is generally divided into the 1D structure (line type) and 2D structure (sandwich shaped). Ma et al. (2019) prepared a dual network hydrogel electrolyte (polyacrylate hydrogel cross-linked by cellulose chains and N,N-methylene-bisacrylamide anchors) and optimized the structure of the zinc and air electrodes to assemble the Zn–air battery with excellent tensile properties. Pan et al. (2019) constructed a sponge-like squeezable Zn–air battery that performed well after 60% compression strain or 500 cycles of repeated compression tests. Li et al. (2018) prepared a 1D knittable Zn–air battery with a diameter of only 1.03 mm through the path, which had an excellent performance of flexibility and charge and discharge.

Table 2 lists more compared performances to provide more meaningful routes for the development of quasi-solid flexible electrolytes for Zn–air batteries. However, it is difficult to get a competent evaluation due to the different battery structure, catalyst, and electrolyte used in recorded works. Therefore, it is necessary to establish a unified evaluation standard for flexible Zn–air battery to evaluate the performance of the corresponding electrolyte better. Furthermore, the composition of the electrolyte in the flexible Zn–air battery is mostly in the “polymer + KOH solution” mode, which leads to the advantages and disadvantages of the aqueous electrolyte mentioned above acting on the quasi-solid electrolyte. At the same time, the combination of RTILs with the polymer may inject new vitality into the safety and stability of Zn–air batteries, but its practical feasibility needs to be verified in the coming future.

**Table 2:** Summary of recently reported quasi-solid flexible electrolyte additive for Zn–air batteries.

**Shape**	**Electrolyte**	**Electrode materials**	**Ionic conductivity (mS cm^**−1**^)**	**Gravimetric energy density (${\text{Wh kg}}_{\text{Zn}}^{-1}$)**	**Power density (mW cm^**−2**^)**	**References**
2D	PAM	Zinc foil//MnO_2_/NRGO@CC	215.6	N/A	105	Miao et al., 2020
2D	Porous PVA + SiO_2_	Zinc plate//Co_3_O_4_@CC	57.3	N/A	80.9	Fan et al., 2019
2D	Porous PVA	Zinc powder//Co_3_O_4_ + LaNiO_3_/NCNT@CC	N/A	581	N/A	Fu et al., 2015
2D	Functionalized biocellulose	Zinc foil//Mn/Fe–HIB–MOF@SS	64	975	194	Shinde et al., 2019
2D	PVP	Zinc powder//FeNC-1@Alkaline member	>10	N/A	250	Wang et al., 2019
2D	PVA	Zinc deposition@Cu film//Ultrathin Co_3_O_4_@CC	N/A	546	N/A	Chen et al., 2017
2D	PANa-cellulose	Zinc@CNT paper//Fe–N–C@CNTP	15-28	930	210.5	Ma et al., 2019
1D	Acrylic polymers	Zinc fiber//NC–Co/CoNx@CF	N/A	N/A	104.0	Guan et al., 2019
1D	PVA@Chiffon band	Zinc wire//Co_3_O_4_/N–rGO@CF	0.33	649	N/A	Li et al., 2018
1D	Gelation	Spiral zinc foil//Fe/N/C	31	N/A	N/A	Park et al., 2015

*CC, carbon cloth; SS, stainless steel; CNTP, CNT paper; CF, carbon fiber*.

## Summary

With the demand for high-power, long life, and flexibility of rechargeable Zn–air batteries, the development of electrolytes meets the opportunities and challenges. Electrolyte, as a critical part of Zn–air battery, has a profound influence on circulation efficiency, power density, and capacity performance. Up to now, alkaline electrolytes are the mainstream because of their excellent ionic conductivity and interfacial properties. However, alkaline electrolytes are susceptible to the effects of carbon dioxide content and relative humidity in the external environment. On the one hand, the suitable type and proportion of additives should be explored to improve the properties of the alkaline electrolyte. On the other hand, RTILs, as an electrolyte for Zn–air batteries, have a high threshold of aging, and its protection and safety for zinc electrodes are apparent. Moreover, the research on quasi-solid flexible electrolyte is more conducive to making portable and flexible Zn–air batteries, which provide that the shortcomings in interface performance and ionic conductivity need to be addressed. Finding the right RTILs and polymers makes sense to improve the performance of the electrolyte.

Furthermore, we think that the three electrolytes mentioned above may be integrated with different features. Suitable electrolyte additives can also promote the application of RTILs and quasi-solid electrolytes in Zn–air batteries, and the combination of RTILs and polymers can also improve the performance of electrolytes. The research on electrolytes should be paid more attention to make Zn–air batteries meet the demand for a new generation of energy storage.

## Author Contributions

All authors listed have made a substantial, direct and intellectual contribution to the work, and approved it for publication.

## Conflict of Interest

The authors declare that the research was conducted in the absence of any commercial or financial relationships that could be construed as a potential conflict of interest.
